# Origin and dynamics of vortex rings in drop splashing

**DOI:** 10.1038/ncomms9187

**Published:** 2015-09-04

**Authors:** Ji San Lee, Su Ji Park, Jun Ho Lee, Byung Mook Weon, Kamel Fezzaa, Jung Ho Je

**Affiliations:** 1 Department of Materials Science and Engineering, X-ray Imaging Center, Pohang University of Science and Technology, 77 Cheongam-Ro, Nam-Gu, Pohang 790-784, Korea; 2 School of Advanced Materials Science and Engineering, SKKU Advanced Institute of Nanotechnology (SAINT), Sungkyunkwan University, Suwon 440-746, Korea; 3 X-ray Science Division, Advanced Photon Source, Argonne National Laboratory, 9700 South Cass Avenue, Argonne, Illinois 60439, USA

## Abstract

A vortex is a flow phenomenon that is very commonly observed in nature. More than a century, a vortex ring that forms during drop splashing has caught the attention of many scientists due to its importance in understanding fluid mixing and mass transport processes. However, the origin of the vortices and their dynamics remain unclear, mostly due to the lack of appropriate visualization methods. Here, with ultrafast X-ray phase-contrast imaging, we show that the formation of vortex rings originates from the energy transfer by capillary waves generated at the moment of the drop impact. Interestingly, we find a row of vortex rings along the drop wall, as demonstrated by a phase diagram established here, with different power-law dependencies of the angular velocities on the Reynolds number. These results provide important insight that allows understanding and modelling any type of vortex rings in nature, beyond just vortex rings during drop splashing.

## Abstract (web summary)

Drop impact on a liquid surface leads to the formation of vortex rings, but this process is still poorly understood due to the lack of effective experimental characterization. Here, Lee *et al*. visualize the process using ultrafast X-ray phase-contrast imaging and follow the dynamics of vortex rings.

## Graphical abstract

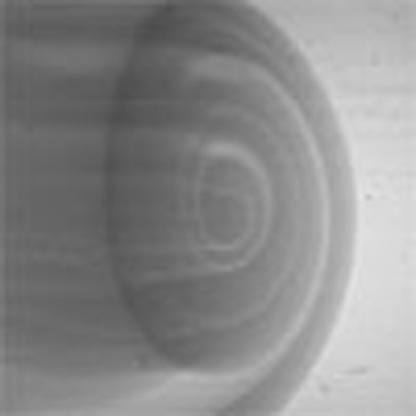

Vortical flow is a main component of turbulence, and it is very commonly observed in nature, such as in winds surrounding a hurricane or a tornado, whirlpools in the wake of boats and paddles and nebulae in space. Specifically, a vortex ring (that is, a torus-shaped vortex) is a fascinating flow phenomenon that is generated when a fluid is rapidly injected into another fluid^1^,^2^, and it is a notable phenomenon in many fields of science^3^,^4^,^5^,^6^. A vortex ring that forms during drop splashing has caught the particular attention of a number of scientists over the past century^7^,^8^,^9^,^10^,^11^,^12^,^13^,^14^,^15^,^16^,^17^,^18^,^20^ ever since the pioneering work by Thomson and Newall was published in the 19th century^7^. This phenomenon is interesting as a result of its fundamental properties in addition to its importance in fluid mixing and mass transport processes.

Figure 1a illustrates the conceptual process for vortex ring formation due to a drop impact. When a liquid drop impinges on a liquid pool, the pool fluid climbs up the drop wall due to surface tension. At the same time, the impact velocity causes the drop fluid not to spread on the pool surface and instead to penetrate the pool. A strong velocity gradient results across the liquid–liquid boundary and then generates azimuthal vorticity that evolves into a vortex ring enveloping the drop^10^,^11^,^12^,^13^,^14^,^15^. Vortex rings are generally known to form when the speed of the impact is sufficiently low or zero^9^,^13^. However, the lack of appropriate visualization has hindered the exact criterion for the vorticity formation and the dynamics of the vortex rings to still remain to be determined even though many efforts have been recently made^18^,^20^,^21^,^22^.

In this study, we elucidate new details of the physical origin for vortex ring formation and its dynamics, and prove and disprove our hypotheses and earlier findings, respectively, based on irrefutable experimental evidence. We also find a row of vortex rings along the drop wall, as demonstrated by the phase diagram established here. Moreover, the vorticity behaviour and the spiral geometry of the vortex rings are characterized in detail.

## Results

### X-ray imaging for vortex ring formation

**Figure 1:**
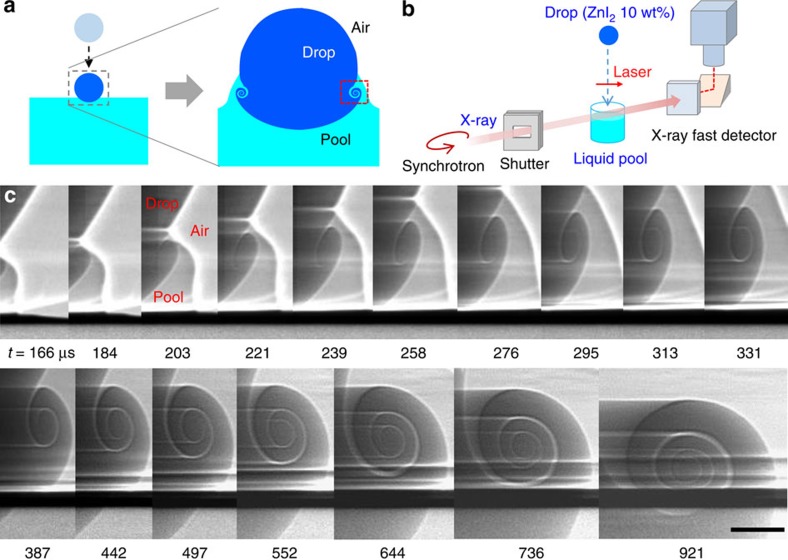
Formation of a vortex ring during drop impact. (**a**) Illustration of the vortex ring formation during drop impact on a pool surface. (**b**) Schematic of X-ray imaging coupled with the experimental set-up for the drop impact. (**c**) Sequential X-ray images (of the red square in **a**) in ethanol drop impact (with diameter ∼1.9 mm) from 80 mm height showing a positive vortical flow (Supplementary Movie 1). The interfacial boundaries between the drop fluid (dark contrast) and pool fluid are clearly resolved in a high temporal resolution. Scale bar, 100 μm long.

To obtain a clear visualization of the vortical flows during drop splashing, we utilized ultrafast X-ray imaging coupled with a drop-impact set-up^20^,^23^. Figure 1b provides in-line projection images of the system with high temporal and spatial resolutions. The refraction-enhanced phase contrast^24^ and the addition of a contrast agent (see the Methods section) enable an unprecedented visualization of the vortical flows during drop impact, as shown in Fig. 1c and in the Supplementary Movie 1. The pool fluid is clearly shown to rapidly climb up the drop wall (166–184 μs) and sharply penetrate into the drop (184–203 μs), forming a spiral. It is remarkable that not only the coiling core, but also the horizontal lines at the crests and troughs that indicate an azimuthally symmetric vortex ring could be clearly resolved at the initial stage of the vortex formation. Such highly vivid imagery enables us to quantitatively analyse the evolution dynamics of the vortices, which has been unreachable through conventional methods.

### Criterion for vortex ring formation

**Figure 2:**
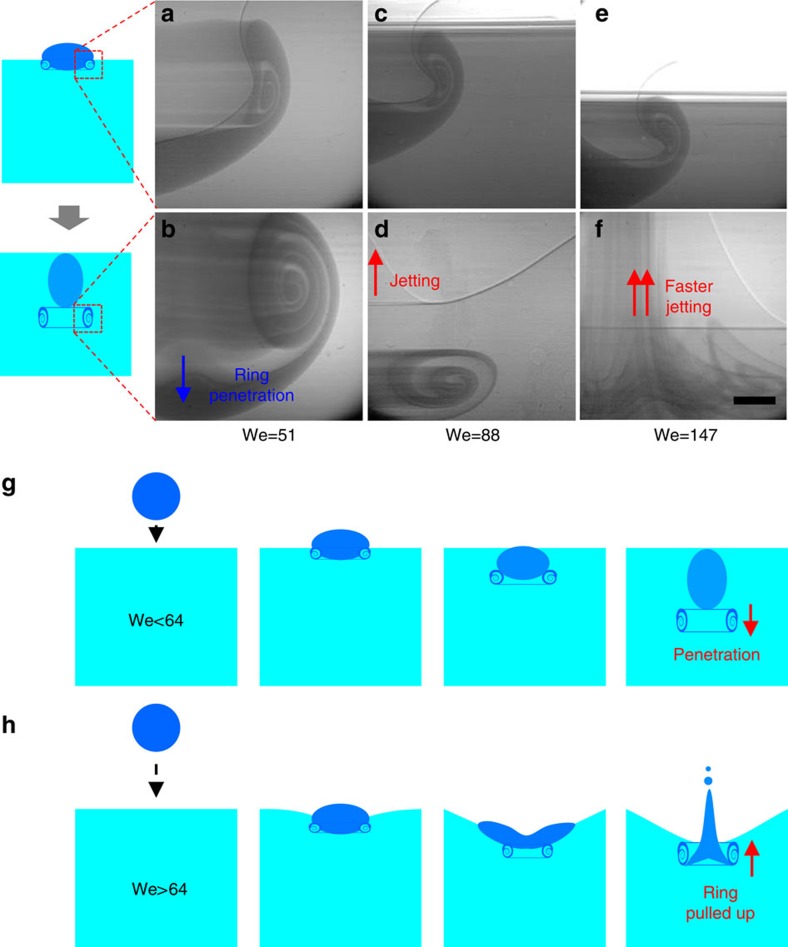
Penetration of the vortex rings. (**a**,**b**) Early (**a**) and late (**b**) stages of vortex ring formation for ethanol drop impact (with diameter ∼1.9 mm) at *H*=35 mm (Supplementary Movie 2). The ring penetrates the pool. (**c**,**d**) Early (**c**) and late (**d**) stages of vortex ring formation for ethanol drop impact at *H*=60 mm (Supplementary Movie 3). The ring is quickly pulled up with a liquid jet. (**e**,**f**) Early (**e**) and late (**f**) stages of vortex ring formation for ethanol drop impact at *H*=100 mm (Supplementary Movie 4). The ring is pulled up much more quickly with a stronger liquid jet. (**g**) Illustration of the vortex ring penetration at We <64. (**h**) Illustration of vortex ring pulled up with a strong liquid jet at We >64. Scale bar, 300 μm long.

First, we solve the long-standing problem of determining when the vortex ring has formed. Over the past two decades, the formation criterion for a vortex ring has been characterized by its Weber number^9^,^13^


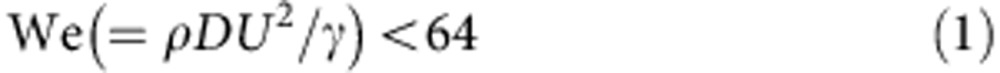


where *ρ* is the liquid density, *D* is the drop diameter, *U* is the drop impact speed and *γ* is the surface tension of the liquid. Our results show that this criterion is not correct and that a vortex ring can actually form, even for a large We (>64), as is clearly seen in Fig. 2a–f and in the Supplementary Movies 2–4. In contrast to the case with a small We (<64) where the drop penetrates the pool, as shown in Fig. 2g–h, the vortex ring is quickly pulled up with the liquid jet for We >64. This result implies that the critical Weber number is only a criterion for ring penetration, irrespective of the ring formation.

**Figure 3:**
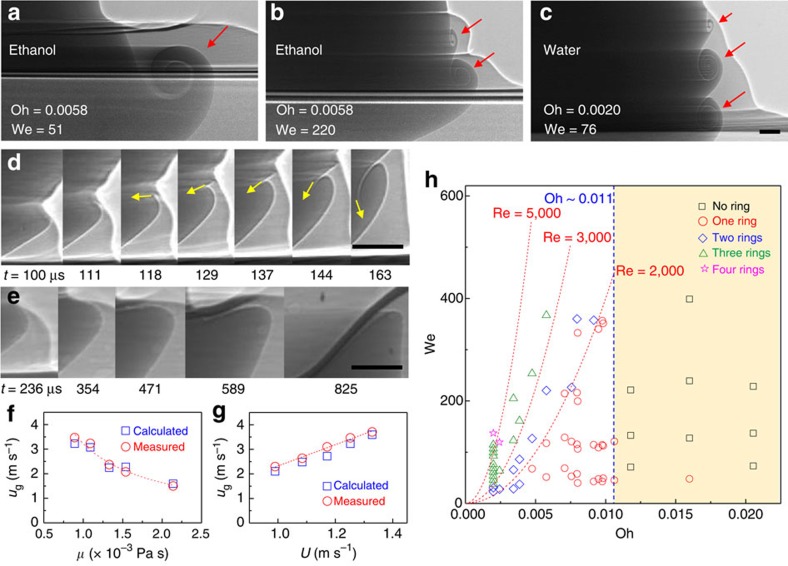
Morphology of vortex rings. (**a**) A single vortex ring formed during ethanol drop (with diameter ∼1.9 mm) impact on the surface at a height of 35 mm (Supplementary Movie 5). (**b**) Double vortex rings formed in ethanol drop impact on the surface at a height of 150 mm (Supplementary Movie 6). (**c**) Triple vortex rings formed in water drop impact on the surface at a height of 100 mm (Supplementary Movie 7). Red arrows clearly show the formation of a single, double and triple rings. (**d**) Ultrafast imaging of vortex formation during the initial stage for water drop (with a diameter of ∼2.9 mm) impact at 80 mm height (Oh∼0.002). Yellow arrows clearly show the penetration of pool fluid into drop fluid. (**e**) Ultrafast imaging of the absence of vortex formation in the impact of a water–glycerol mixture (60 wt% of glycerol) drop (with a diameter of ∼2.8 mm) at 80 mm height (Oh∼0.02). (**f**) Comparison of the measured group velocities of the capillary waves with the values calculated for water–glycerol mixtures with a different liquid viscosities (*μ*) at an 80-mm impact height. The dotted line is the best fit for the values measured with allometric scaling: *u*_g_∝*μ*^–0.98^. (**g**) Comparison of the measured group velocities of the capillary waves with the values calculated for water at different impact velocities (*U*). The dotted line is the best fit for the values measured with allometric scaling: *u*_g_∝*U*^1.13^. (**h**) Phase diagram for the multiplicity of the vortex rings in terms of Oh and the Weber number (We). Three to ten experimental repetitions were performed for each data point to verify the repeatability. Scale bar, 100 μm long.

We have found that the criterion to determine whether a vortex ring forms is the Ohnesorge number, which relates the viscous forces to the inertial and capillary forces as Oh=*μ*/(*ρDγ*)^0.5^ (with *μ* as the dynamic viscosity of liquid), rather than the Weber number. As shown in the phase diagram of Fig. 3h, an investigation of a wide range of liquids using water, glycerol and ethanol mixtures shows that the vortex rings formed only for Oh <0.011. Furthermore, we find that a row of vortex rings forms along the drop wall, which correspond to single, double and triple rings, respectively, as shown in Fig. 3a–c (see red arrows) (Supplementary Movies 5–7). When the Reynolds number increases (Re=*ρDU*/*μ*=We^1/2^/Oh), we specifically observe single rings at Re <2,000, double rings at Re >2,000, triple rings at Re >3,000 and quadruple rings at Re >5,000 (Fig. 3h).

Here the Oh dependency of the ring formation is explained to be a result of the capillary waves that are known to develop azimuthally at the impact moment and subsequently travel along the drop wall^25^. When the capillary waves propagate about one wavelength, the pool fluid penetrates sharply into the drop as a result of the momentum that is transported by the waves, followed by the vortices coiling, as clearly shown with yellow arrows in Fig. 3d. However, for a high Oh (>0.011), the onset of the vortices is suppressed by a strong viscous dissipation of the momentum transfer, as shown in Fig. 3e.

The Re dependency of multiple vortex rings shown in Fig. 3a–c,h can be explained to be a result of the capillary waves as well. The wave velocities measured from the X-ray images are comparable to the group velocities of the capillary waves, *u*_g_=1.5(2*πγ*/*ρλ*)^1/2^, where *λ* is the wavelength measured over a wide range of Oh (Fig. 3f) and impact speeds (Fig. 3g). The dependencies of the wave velocity on the hydrodynamic factors are roughly estimated as *u*_g_∝*μ*^–1^ and *u*_g_∝*U*, as fitted in Fig. 3f,g, respectively. These suggest that the long-range energy transfer by capillary waves is more favourable in a large Re with *u*_g_∝*U*/*μ* ≡ Re, which explains the increase in the number of the rings with Re in Fig. 3h. In fact, the formation of a row of multiple vortex rings is observed in the simulation^26^ and with X-ray imaging experiments^20^. Here, we are the first to find the criteria in terms of dimensionless numbers to determine the number of vortex rings and to roughly explain the hydrodynamics of the phenomenon.

### Hydrodynamics of the vortex rings

**Figure 4:**
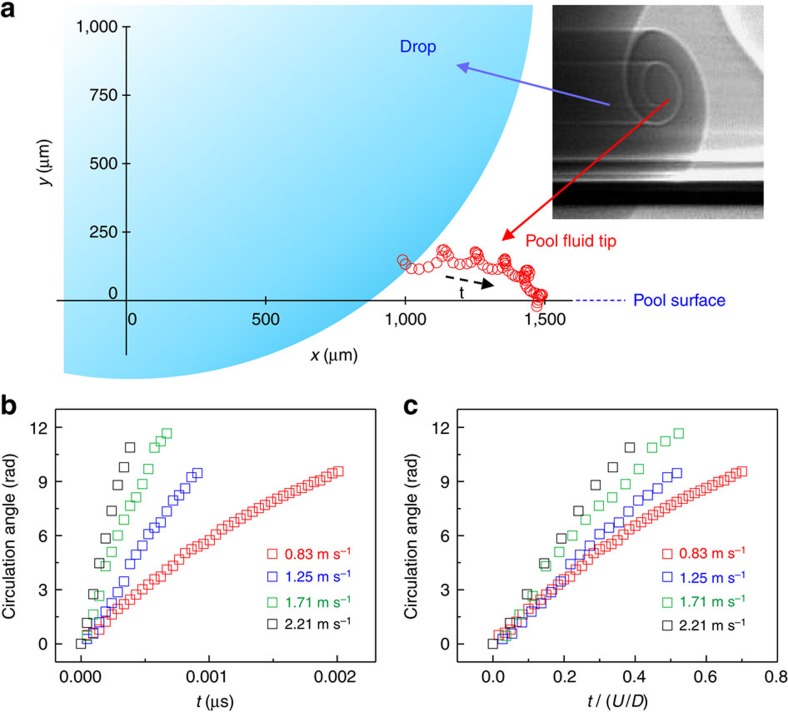
Time evolution of the vortex ring formation. (**a**) Tracking the coiling vortex core with time in water drop impact. The *x*–*y* coordinates are set to the real size of the impacting drop. *x*=0 is the centre of the drop and *y*=0 is the top of the pool surface. (**b**) Circulation angle of the vortex core as a function of time for the water–ethanol (3:7 in mass fraction) drop impact with different impact speeds. (**c**) Circulation angle of the vortex core as a function of time normalized with the characteristic time *U*/*D* with the same data in **b**.

**Figure 5:**
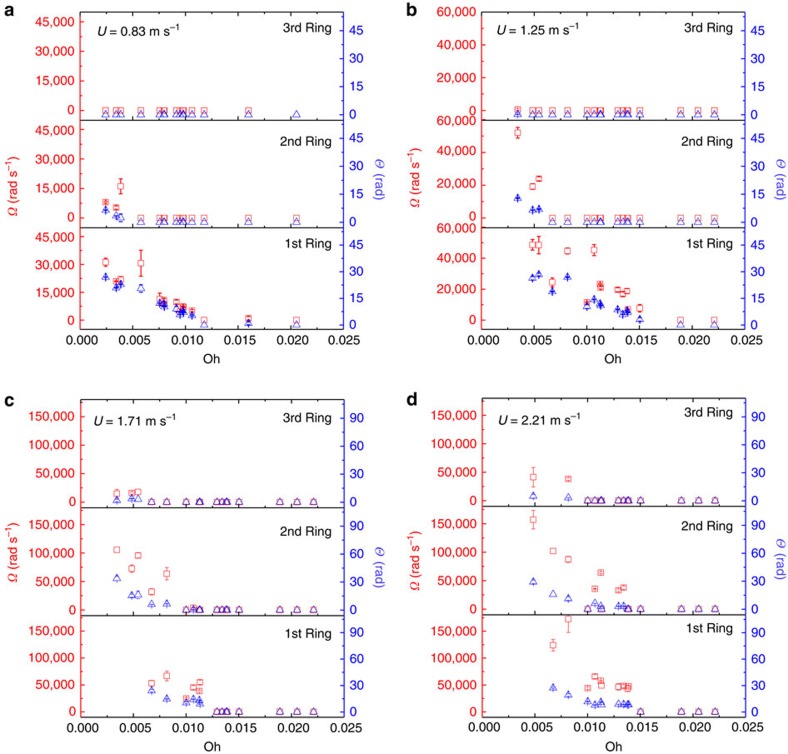
Circulation measurement of vortex rings. Average angular velocity Ω (red squares corresponding to the left axis) and total circulation angle Θ (blue triangles corresponding to the right axis) measured for three subsequent rings as a function of Oh. (**a**) Measurement at an impact speed *U*=0.83 m s^−1^. (**b**) Measurement at an impact speed *U*=1.25 m s^−1^. (**c**) Measurement at an impact speed *U*=1.71 m s^−1^. (**d**) Measurement at an impact speed *U*=2.21 m s^−1^.

**Figure 6:**
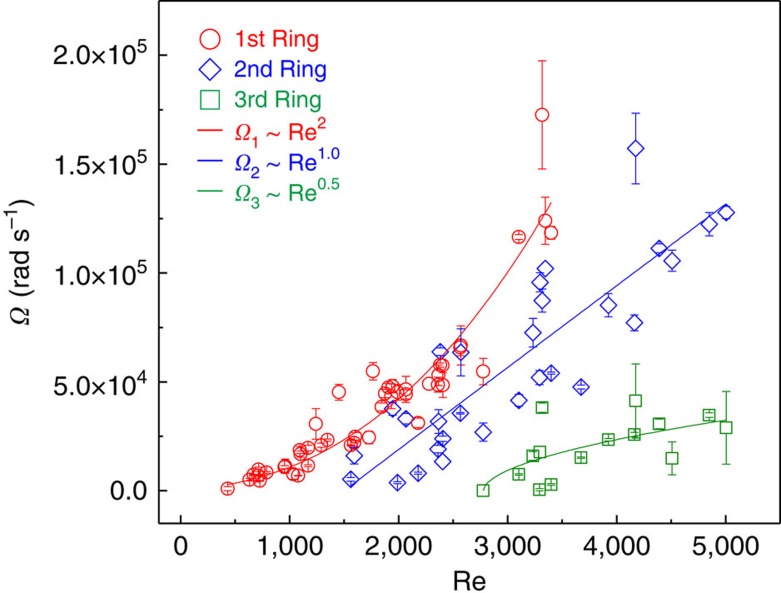
Dynamics of vortex rings. Average angular velocity of spirals (Ω) is measured for three subsequent rings as a function of the Reynolds number (Re). The best fit for the three rings shows different Re dependencies. Ω_1_∝Re^2^ for the first ring, Ω_2_∝Re^1.0^ for the second and Ω_3_∝Re^0.5^ for the third.

For the first time, we have measured the circulation dynamics of the vortices, specifically, the time-dependent circulation angles of the vortex cores, as shown in Fig. 4a,b. Figure 4c is rescaled in time and shows that the circulation dynamics are not simply described by *D*/*U*, as previously reported in the literature^15^,^27^. As seen for the total circulation angle Θ and average angular velocity Ω (obtained by dividing the total angle by the time), which are measured over a wide range of impact conditions (Fig. 5), Θ and Ω consistently decrease as Oh increases. Figure 6 is plotted again as a function of Re, showing that the angular velocity of spiral increases as Re increases in a different manner with ring order. Specifically, Ω_1_∝Re^2^ for the first ring, Ω_2_∝Re^1^ for the second and Ω_3_∝Re^0.5^ for the third, which is largely different from the previous simple prediction Ω∝Re^0.5^ (refs 17, 26).

**Figure 7:**
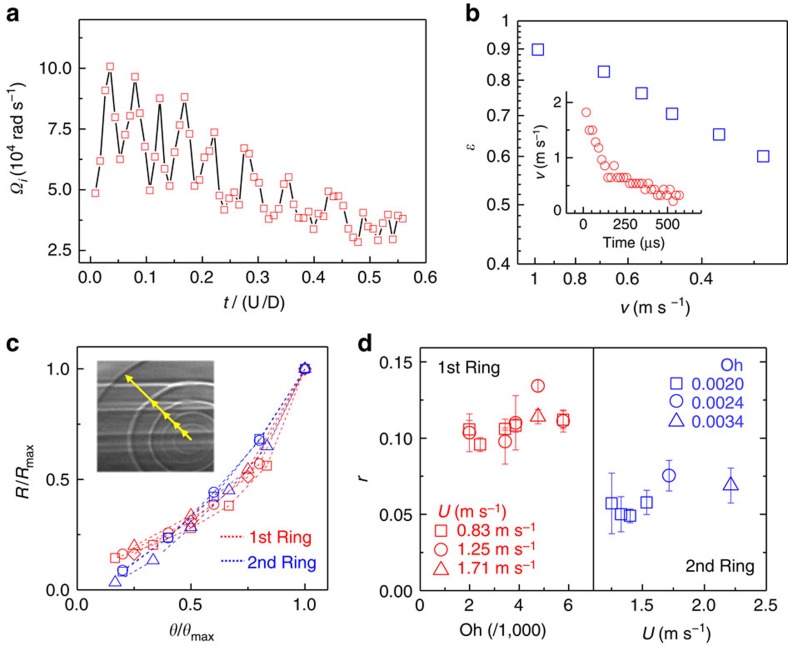
Geometry of the vortex streamlines. (**a**) Variation in the instantaneous angular velocity plotted with time and rescaled with the characteristic timescale *τ*=*U*/*D* for water drop impact at a height of 80 mm. The angular velocity shows a substantial oscillatory decrease over time. (**b**) The eccentricity of the vortex streamlines as a function of the expansion speed of the vortex ring (*v*), as measured from the same images as **a**. Inset, the expansion speed of the vortex ring measured with time. (**c**) Measurement of the spiral radii, as shown in the inset, as a function of the circulation angle (*θ*) normalized with the accumulated angle (*θ*_max_) at the outermost curve. The red data points are for the first ring in the drop impact at *H*=35 mm with different liquids (squares for Oh=0.0020, circles for Oh=0.0024, triangles for Oh=0.0034 and diamonds for Oh=0.0039). The blue data points are for the second ring in the drop impact with water (Oh=0.0020) at different impact heights (squares for *H*=80 mm, circles for *H*=90 mm and triangles for *H*=100 mm). (**d**) Best-fit values for the *r* of the vortex rings. (Left) *r* values of the first rings plotted with Oh for the given impact speed (*U*). (Right) *r* values of the second rings plotted with *U* for the given Oh.

How does the angular velocity change over time? In general, the vorticity decreases with time as a result of the viscosity^17^,^26^. Here we have measured the instantaneous angular velocity of the vortex ring (the angular velocity of the circulating vortex tip at the centre of the spiral) as Ω_i_=2*κq*, with *κ* as the interface curvature and *q* as the tangential flow velocity^11^. Interestingly, we find an apparent oscillatory decrease in the angular velocity with time, as shown in Fig. 7a. In particular, the strong oscillation during the initial stage is conceivably a result of an elliptical deformation of the vortex (Fig. 3d), due to the high-speed expansion of the vortex ring on the pool surface from the spreading of the impacting drop. The eccentricity of the vortex *ɛ*, shown in Fig. 7b (obtained from the same image data for Fig. 7a), decreases as the expansion speed (*v*) decreases, that is, with an increasing time (inset of Fig. 7b), which explains the weakening of the oscillation in a later stage.

We were able to measure the spiral geometry of the vortex streamlines, specifically the radius of each spiral layer for the first and the second rings (Fig. 7c). The vortex rings show logarithmic spirals similar to those in various vortical systems in nature, such as a nautilus hemishell, tornados and galaxies^28^, and these can be represented in a polar coordinate system (*R*, *φ*) as *R*∼*R*_0_e^*rφ*^, where *R*_0_ is the initial radius and *r* is the growth-rate coefficient. The best-fit values of *r*, which are estimated for the two subsequent rings (*r*_1_ and *r*_2_) in various impact conditions (Fig. 7d), are almost invariant in each ring with *r*_1_ almost twice that of *r*_2_ (*r*_1_∼0.12±0.02 and *r*_2_∼0.06±0.01) regardless of the liquid viscosity or the drop impact speed. The *r* values are notably much smaller than those of typical logarithmic spirals in nature. For example, *r*∼0.18 for a nautilus shell,∼0.21 for the Milky Way and ∼0.31 for typical golden spirals^28^.

### Relation of the vortex ring and the liquid jet formation

**Figure 8:**
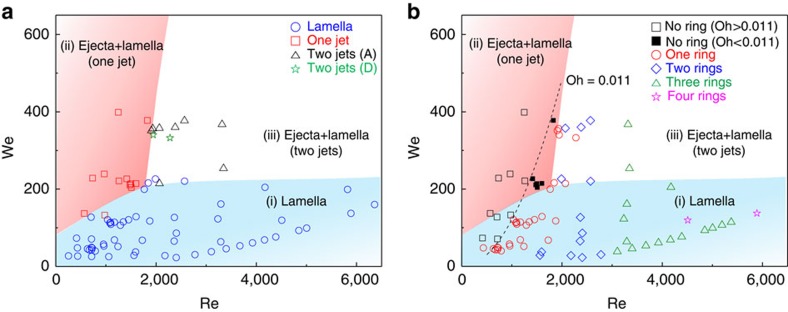
Relation between the jetting phenomena and the vortex ring morphologies. (**a**) Phase diagram for a number of jets during drop impact as a function of We and Re. The jetting phenomena are indicated with symbols (blue circles for capillary-induced lamella, red squares for a merged single jet comprising lamella and ejecta, black triangles for separate lamella and ejecta where the latter is finally reabsorbed into liquid and green stars for separate lamella and ejecta where the latter finally dissociates into tiny droplets). Each region with (i) only lamella, (ii) a single jet comprised of the ejecta and lamella and (iii) two jets of the ejecta and lamella is coloured with blue, red and white, respectively. (**b**) Phase diagram for a number of vortex rings in the drop impact as a function of We and Re. The number of vortex rings is indicated with the symbols shown in the legend. The coloured regions are the same as those in **a**. It is clearly shown that no vortex ring is generated in region ii (single jet comprising lamella and ejecta) even if the Ohnesorge number is smaller than the critical value of 0.011. (The error range of each data point is much smaller than its symbol size.).

**Figure 9:**
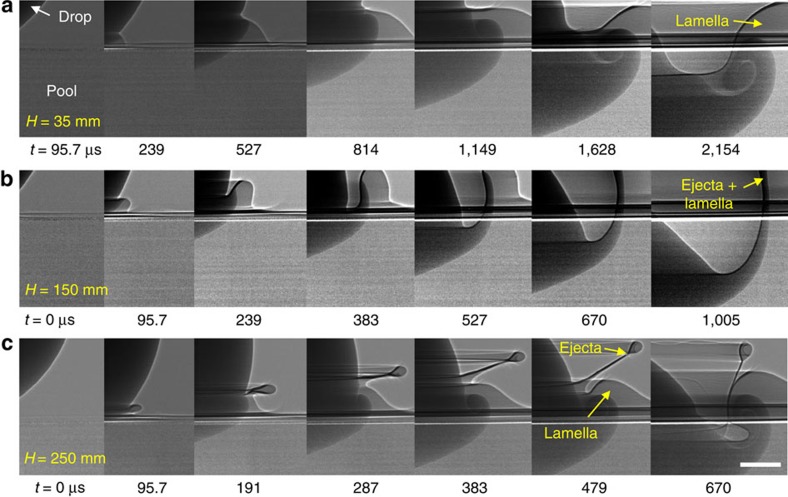
X-ray images of jetting phenomenon in drop impact. Here water–ethanol (4:6 in mass fraction) drops are used. (**a**) Impact at *H*=35 mm (*U*=0.83 m s^−1^) with We=50 and Re=724. Only a lamella is formed, and the vortex ring is generated within the lamella. (**b**) Impact at *H*=150 mm (*U*=1.71 m s^−1^) with We=214 and Re=1,498. An ejecta is merged with the lamella, suppressing the formation of a vortex ring. (**c**) Impact at *H*=250 mm (*U*=2.21 m s^−1^) with We=356 and Re=1,934. The drop diameter is 2.3 mm. An ejecta and a lamella separately emerge, and the vortex ring is formed within the lamella. Scale bar, 300 μm long.

We finally discuss a relation between the vortex ring formation and the jetting phenomena. Two kinds of jets were ejected after a drop impact on a liquid pool^17^,^29^,^30^,^31^. The ejecta is an inertia-induced thin liquid sheet that emerges rapidly from the neck between the drop and the pool surface, and the lamella is a thicker capillarity-induced liquid sheet that propagates outwards from the impact site. As shown in the phase diagram in Fig. 8a^31^, three regimes exist for the jetting phenomena, including the (i) lamella emerging at a low We (Fig. 9a), (ii) the ejecta merging with the lamella into one jet at a high We and a low Re (Fig. 9b) and (iii) the ejecta and lamella developing separately at a high We and high Re (Fig. 9c). These three regimes are shown in different colours, and we plotted the number of the vortex rings in terms of the Re and the We in Fig. 8b. For regimes i and iii, the vortex rings form robustly as long as Oh <0.011. In regime iii, we note that the vortex ring forms within the lamella that has developed separately from the ejecta (Fig. 9c). In regime ii, however, the vortex ring never forms despite having a small Oh (<0.011), indicating that the ejecta, once merged with the lamella, suppresses the ring formation. This suggests that the lamella should not be merged with the ejecta as an additional condition for vortex ring formation.

## Discussion

In this study, we have experimentally revealed the dynamics of vortex rings in a drop impact that is unresolvable with optical imaging, and we have therefore mostly relied on theoretical studies. In contrast with recent studies on the vortex rings^18^,^20^,^26^, we have made a great advance in addressing issues that had been unsolved for a long time. The criterion for vortex ring formation, the ring morphology dependence on the hydrodynamic conditions, the vorticity or energy of the vortex rings and the relation with an external splashing shape were investigated using a novel ultrafast X-ray imaging technique. This study therefore offers substantial insight for further analytical, numerical and experimental work on vortex ring dynamics in fluid mechanics.

## Methods

### X-ray imaging

Ultrafast X-ray imaging experiments were carried out using the XSD 32-ID beamline of the Advanced Photon Source, Argonne National Laboratory. An intense white (full energy spectrum) X-ray beam with a peak irradiance of ∼10^14^ ph s^−1^ mm^−2^ per 0.1% bandwidth (bw) was used to conduct the high imaging speed. The detector system (Fig. 1b) is composed of a fast scintillator (LuAG:Ce, decay time ∼50 ns) and a right-angle mirror coupled to a high-speed camera (Photron Fastcam SA 1.1) via a long-working distance microscope objective. The detector system is synchronized to the X-ray pulses to enable the direct visualization of the ultrafast dynamics of the liquid–air and liquid–liquid interfaces at up to ∼270,000 frames per a second, with a 472-ns exposure time for each frame.

### Experimental set-up

Liquid drops were dispensed from a 26-G syringe needle (outer diameter ∼0.46 mm and inner diameter ∼0.26 mm) at heights from 30 to 300 mm. A laser triggering system was installed to sense the falling drop and to trigger the camera and the fast shutter to take the images. The liquid pool was prepared in a cylinder made of Kapton with a diameter of 20 mm and a depth of 50 mm.

### Materials

The liquids were prepared by mixing water, ethanol and glycerol with various compositions. For all experiments, the liquids for the falling drop and the pool were prepared with identical compositions, except for the addition of zinc iodide (10 wt%) in the drop as a contrast agent. The addition of the small fraction of zinc iodide can increase the X-ray absorption and can therefore improve the image contrast between the drop and the pool fluids. This small concentration of zinc iodide (∼0.006 M for water and ∼0.014 M for ethanol) can slightly increase the viscosity of the fluid (∼1% for water and ∼3% for ethanol)^32^, but we assume that this change does not have a significant influence on the general trend of the hydrodynamics.

## Additional information

**How to cite this article:** Lee, J. S. *et al*. Origin and dynamics of vortex rings in drop splashing. *Nat. Commun.* 6:8187 doi: 10.1038/ncomms9187 (2015).

## Supplementary Material

Supplementary Movie 1 X-ray imaging of vortex ring formation during the impact of an ethanol drop onto an ethanol pool (relevant to Fig. 1c).

Supplementary Movie 2 X-ray imaging of vortex ring formation during an ethanol drop impact at H = 35 mm showing a ring penetration (relevant to Figs. 2a and 2b).

Supplementary Movie 3 X-ray imaging of vortex ring formation during an ethanol drop impact at H = 60 mm showing a ring quickly pulled by a liquid jet (relevant to Figs. 2c and 2d).

Supplementary Movie 4 X-ray imaging of vortex ring formation for an ethanol drop impact at H = 100 mm showing a ring much more quickly pulled by a liquid jet (relevant to Figs. 2e and 2f).

Supplementary Movie 5 X-ray imaging during an ethanol drop impact at H = 35 mm showing the formation of a single vortex ring (relevant to Fig. 3a).

Supplementary Movie 6 X-ray imaging during an ethanol drop impact at H = 150 mm showing the formation of double vortex rings (relevant to Fig. 3b).

Supplementary Movie 7 X-ray imaging during a water drop impact at H = 100 mm showing the formation of triple vortex rings (relevant to Fig. 3c).
